# From Aspiration to Action: Aligning the Pandemic Agreement with Equity in Vaccine Access; A Response to Recent Commentaries

**DOI:** 10.34172/ijhpm.9192

**Published:** 2025-06-11

**Authors:** Luciana Correia Borges, Henrique Zeferino de Menezes, Eric Crosbie

**Affiliations:** ^1^School of Public Health, University of Nevada, Reno, Reno, NV, USA.; ^2^Department of International Relations, Federal University of Paraiba, João Pessoa, Brazil.; ^3^Ozmen Institute for Global Studies, University of Nevada, Reno, Reno, NV, USA.

 The commentaries by Gleeson et al,^1^ Ortiz-Millán,^2^ Lexchin,^3^ and Kumar Chattu et al^4^ on our article^5^ offer critical reflections that reinforce and expand our argument that disparities in COVID-19 vaccine rollouts expose structural inequities in global health governance, driven by pharmaceutical profit prioritization and compounded by voluntary compliance frameworks. The recently concluded negotiations by the World Health Organization’s (WHO’s) Intergovernmental Negotiating Body on the Pandemic Agreement—approved by 191 countries and to be considered at the 78th World Health Assembly—are cited across the commentaries as a pivotal moment for institutionalizing solidarity and corporate accountability. Building on our analysis and these contributions, we revisit the Agreement’s challenges and opportunities, assessing how scholarly critique can inform enforceable reforms.

 Our article analyzed how high-income countries achieved nearly 50% vaccine coverage by June 2021, while low-income countries remained below 1%, despite the efforts of COVAX—a global initiative co-led by Gavi, WHO, and the Coalition for Epidemic Preparedness Innovations to ensure equitable vaccine distribution. We argued that this disparity reflected not just logistical constraints such as production delays but systemic inequities in a health architecture where public health priorities were treated as secondary to commercial interests. Voluntary norms and corporate discretion failed to safeguard equitable access, highlighting the need for a binding international framework to protect the right to health during global health emergencies.^1^

 Synthesizing the commentaries, we observe strong consensus on two critical points: voluntary mechanisms are inadequate to guarantee equitable vaccine access, and systemic reforms, not ad hoc responses, are necessary. However, differences emerge in emphasis. Gleeson et al^1^ focus on the treaty’s language weaknesses, warning that equity goals may remain aspirational without enforceable obligations. Ortiz-Millán^2^ advances the ethical dimensions, asserting that vaccines are global public goods requiring distributive justice frameworks. Lexchin^3^ deepens the critique, historicizing pandemic profiteering as a symptom of entrenched exclusionary pharmaceutical practices. Kumar Chattu et al^4^ broaden the analytical lens, emphasizing the need for stronger political prioritization, multilateral diplomacy, and corporate accountability. This synthesis highlights the intertwined responsibilities of corporations, states, and international institutions in advancing equitable pandemic governance.

 The WHO Pandemic Agreement, finalized in April 2025, directly intersects with these critiques. While initial ambitions aimed at transformative reform, negotiations diluted key provisions on supply chain transparency, technology transfer, and equitable intellectual property practices. Nonetheless, notable gains remain: pharmaceutical companies participating in the WHO Pathogen Access and Benefit-Sharing (PABS) system must allocate 20% of pandemic-related production to the WHO, with donations prioritized for low-income countries.^6^ This formalization of benefit-sharing partially addresses historic inequities, even as deeper structural reforms remain necessary.

 The Agreement partially addresses the concerns raised by the commentaries through structured, albeit limited, legal commitments. In response to Gleeson and colleagues’ concerns, the final text removes the term “voluntary” concerning technology transfer; however, it still only requires Parties to “promote and facilitate” such transfer on mutually agreed terms—falling short of the binding obligations initially envisioned.^6^ Yet Articles 10 and 11 introduce structured commitments for diversifying manufacturing capacity, while Article 20 establishes sustainable financing mechanisms crucial for operationalizing equity. The document uses the phrase “each Party shall strengthen” regulatory frameworks, marking a move toward firmer, though still incomplete, legal commitments compared to past soft-law approaches.

 The Agreement also echoes the values emphasized by the other commentaries. Ortiz-Millán’s call for distributive justice is reflected in Article 11’s promotion of non-exclusive licensing and WHO-led technology pools. Lexchin’s critique aligns with Article 12’s PABS system, ensuring that pathogen-sharing yields public benefits rather than reinforcing monopolistic practices. Kumar Chattu and colleagues’ advocacy for robust governmental action finds resonance in Articles 3 and 19, which institutionalize whole-of-government and whole-of-society approaches to pandemic governance.^6^

 Despite these advancements, core challenges remain. The tension between political feasibility and legal enforceability is visible throughout the Agreement. Sovereignty concerns and industry influence constrained the binding strength of equity measures. Thus, while the Agreement significantly advances in codifying global solidarity, it demands vigilant implementation, robust political commitment, and iterative strengthening through future diplomatic processes.

 In conclusion, the commentaries collectively reaffirm the urgency of transforming pandemic governance structures. They strengthen our original call for enforceable frameworks that place equity and public health above market imperatives. The WHO Pandemic Agreement represents a foundational, though incomplete, step in this direction. If adopted during the 78th World Health Assembly, its success will hinge on sustained pressure from states, multilateral bodies, and civil society to ensure that global health responses are driven by solidarity, justice, and universal access.

## Ethical issues

 Not applicable.

## Conflicts of interest

 Authors declare that they have no conflicts of interest.
